# Laser Resurfacing for the Management of Periorbital Scarring

**DOI:** 10.20517/2347-9264.2020.77

**Published:** 2020-11-20

**Authors:** Nathan Pirakitikulr, John J. Martin, Sara T. Wester

**Affiliations:** 1 Division of Oculofacial Plastic and Reconstructive Surgery, Department of Ophthalmology, Bascom Palmer Eye Institute, University of Miami-Miller School of Medicine, Miami, FL 33136, USA; 2 Private practice, Coral Gables, FL 33134, USA

**Keywords:** Periorbital scarring, ectropion, laser resurfacing, laser assisted drug delivery

## Abstract

Laser (light amplification by the stimulated emission of radiation) skin resurfacing is currently one of the most widely adopted technologies in facial rejuvenation. While most often used for aesthetic purposes, lasers also have applications in the management of scars. Since the introduction of the CO_2_ laser for skin rejuvenation in the 1990s, the last three decades have seen significant growth in the number of laser devices available to the physician. More recently, promising alternatives to light-based resurfacing technologies have emerged that include radiofrequency and intense focused ultrasound. To help the physician navigate the most current laser technologies as they apply to periocular scars, this review discusses the available treatment modalities, pre-treatment assessment of periorbital scars, treatment selection, and reported outcomes and complications. The recommendations described herein are based on published literature and the authors’ experience in an academic oculoplastics practice.

## INTRODUCTION

Laser skin resurfacing is an important adjunct in the management of many types of periorbital scars. Skin in this region is prone to photoaging, telangiectasias, erythema, and hypertrophy. Scars arising from prior surgery, trauma, or inflammation are highly visible and may ultimately compromise the mechanical function of the eyelids, thereby causing damage to the ocular surface. Initially, periorbital scars are most often managed conservatively with mechanical massage or medically with topical and intralesional corticosteroids and antimetabolites such as 5-fluorouracil^[1]^. Lasers can be used as an alternative or in combination with some of these therapies^[2]^. Lasers can help soften scar tissue through controlled thermal damage to the skin to promote collagen remodeling^[3,4]^. In addition, lasers can aid in topical drug delivery by increasing skin permeability, which helps distribute and increase the penetrance of topically applied medications^[4]^. By selectively targeting specific chromophores, lasers can also be used to address dyspigmentation^[5]^. Complications are rare with proper preoperative assessment and technique, but the susceptibility of the eye to laser damage warrants special precautions. In this review, we present a general approach to treating periorbital scars with laser. Recommendations are based on the authors’ clinical practice and a review of the PubMed-indexed literature published within the last 30 years. Sources include systematic reviews, meta-analyses, and clinical trials, which are cited accordingly throughout the text.

## PRINCIPLES OF PERI-OCULAR LASER SKIN RESURFACING

A wide range of lasers has been used to treat the periorbital tissue [Table 1]^[6,7]^. Lasers used for skin resurfacing are defined by their lasing medium and emission wavelength, and further categorized based on whether the superficial epidermis is removed during treatment. Ablative lasers, which include CO_2_, Erbium:YAG, and Erbium:yttrium-scandium-gallium-garnet (Er:YSGG) lasers, were the first lasers to come to market and target both the dermis and the overlying epidermis. These lasers can be very effective; however, they also carry a greater risk of causing scarring and hyperpigmentation, particularly in patients with higher Fitzpatrick skin types. In contrast, non-ablative lasers do not cause thermal damage to the overlying epidermis. Examples include Erbium:glass, diode, Nd:YAG, alexandrite, ruby, pulsed dye (PDL), and potassium titanyl phosphate.

Both ablative and non-ablative lasers can be fractionated. Fractionation divides a single laser beam into thousands of microscopic beams of light that generate columns of treated tissue and leave intervening skin untouched. This allows treatment depth to be safely increased and creates deeper channels for topical drug delivery, as discussed below^[8]^. With the thermal energy distributed over a larger surface area, there is also a lower risk of overtreatment^[9]^. While maintaining similar efficacy, fractionation has made ablative lasers in particular much safer because, by leaving small areas of tissue untreated, areas of ablated epidermis re-epithelialize more rapidly^[10]^.

Other light-based therapies such as intense pulsed light (IPL) or BroadBand Light (BBL)™ emit a spectrum of light rather than a single wavelength. These are also used for non-ablative skin treatment in the periocular region, but care must be taken with these light-based therapies as the risk of ocular damage is high without proper precautions^[11]^.

Light emitted from all lasers can be delivered in a continuous wave form or, more commonly, as long-pulsed, nanosecond (also referred to as Q-switched) or picosecond pulses. By selecting a wavelength that is preferentially absorbed by a target chromophore, applying enough energy to cause thermal destruction, and setting a pulse duration shorter than the target’s thermal relaxation time, tissues can be targeted at precise depths for treatment with minimal surrounding damage^[12]^.

As with laser use in other areas of the face, there are few true absolute contraindications to the use of periocular laser, but these are important considerations when determining appropriate timing of treatment. These contraindications apply more directly to the use of ablative lasers due to the induced loss of epidermis. Examples include oral retinoid use within the last six months and active skin infections^[13]^. Other relative contraindications include history of poor wound healing, personal history of abnormal scarring or keloids, smoking, or diabetes. Patients with a history of herpetic lesions should be started on prophylactic doses of antivirals prior to laser treatment. Practices vary by practitioners as to timing and dose, and many practitioners advocate prophylactic treatment in all patients undergoing ablative laser resurfacing regardless of prior history.

## ASSESSMENT OF PERIOCULAR SCARS

For the purposes of selecting the appropriate treatment, whether that involves a laser modality, pharmacologic therapy, a combination or expectant observation, the pertinent factors to consider are whether the scar is under tension, if and how the scar negatively impacts the eye, and what are the scar characteristics (form, depth, and age). Scars that cause skin contracture or are under significant tension may affect the opening and closure of the eyelid, and therefore require prompt treatment to avoid permanent damage to the eye. Scars that may be observed or treated less urgently include hypo- and hyperpigmented lesions, hypertrophic raised lesions, and erythema that do not impact eyelid function. Many of these scars can respond well to laser resurfacing provided that the appropriate chromophore and tissue depth can be safely targeted. For instance, most pigmented or vascular scars do best with PDL and IPL/BBL™^[14]^.

In the periorbital region, the thickness of epidermis ranges from 130 to 202 μm and from 215 to 969 μm for dermis [Table 2]^[15]^. Eyelid skin is among the thinnest found on the body The thickest skin in the periocular region is found on the forehead and cheek. Ablative CO_2_ lasers can target tissue up to 2 mm in depth, deeper than is necessary for periorbital cosmetic skin resurfacing. With fractional CO_2_ lasers, the treatment depth can be controlled by adjusting treatment power and spot size^[16]^. This is useful for safely treating large hypertrophic scars, periorbital rhytids, and laxity Non-ablative lasers such as Nd:YAG are limited to depths less than 1 mm, which is sufficient to treat most superficial scars and limits the risk of adverse outcomes.

**Figure 1.**
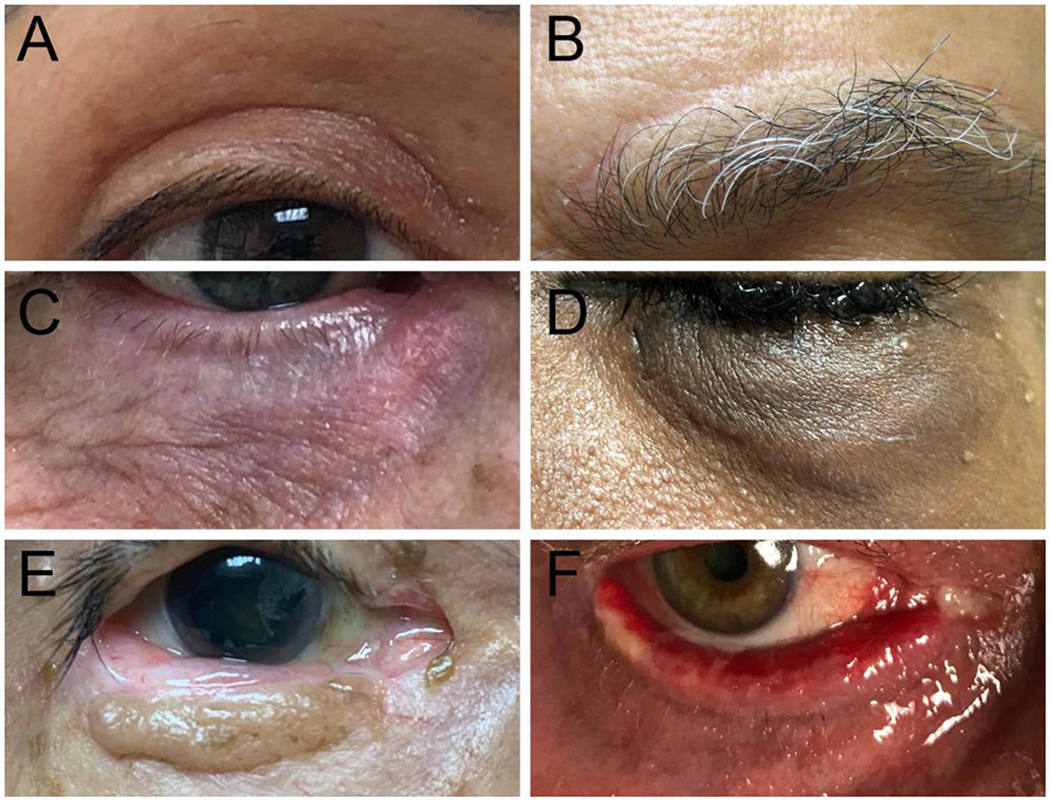
Post-surgical scars occurring in the periorbital region: (A) a well-concealed surgical incision within the lid crease following upper eyelid blepharoplasty; (B) a surgical scar above the brow following a direct browplasty procedure; (C) prolonged ecchymosis and dyspigmentation of the lower eyelid skin following a canalicular laceration repair; (D) hyperpigmentation of eyelid skin following complex repair of a lower eyelid avulsion; (E) dyspigmentation and hypertrophy of skin following a Hughes tarsoconjunctival flap and bipedicle flap to reconstruct the lower eyelid years after excision of a basal cell carcinoma by an outside provider; and (F) lower eyelid ectropion in a patient with an underlying inflammatory dermatosis

Post-surgical scars present a unique challenge as patients may associate the presence of scars with the overall surgical outcome^[17,18]^. Many procedures performed by oculoplastic surgeons serve both functional and cosmetic purposes (e.g., blepharoplasties, ptosis repair, and browlifts). Smaller surgical scars such as from blepharoplasties may hide well under skin folds or cilia [Figure 1A], but scarring at exposed incision sites can be aesthetically displeasing, as well as cause itchiness or pain. For instance, the direct browplasty, which is used to treat moderate to severe degrees of brow ptosis in select cases for whom other approaches are not possible, can leave a visible scar immediately above the brow that is displeasing to patients [Figure 1B].

Scars that arise from reconstructive eyelid surgeries are often unpredictable and range from prolonged ecchymosis [Figure 1C] and mild hyperpigmentation [Figure 1D] to hypertrophy and contractures leading to malposition of the eyelid [Figure 1E]. Earlier intervention is indicated in cases of eyelid malposition causing severe ectropion, where the ocular surface may quickly become compromised. Specific procedures more prone to causing eyelid malposition include skin flaps, full thickness skin grafts, and lower eyelid blepharoplasties where excess skin is excised. Scars that deform the eyelid require prompt attention as eversion of the eyelid margin exposes the conjunctiva and cornea and leads to chronic ocular irritation, redness, and corneal compromise that can ultimately result in corneal ulcers or even corneal melt. Similarly, severe scars are seen with trauma, radiation, chemical injury, thermal injuries, and chronic inflammation [Figure 1F], and they have been found to benefit from laser resurfacing^[2,19]^.

## CHOOSING THE APPROPRIATE TIMING FOR TREATMENT

Choosing the appropriate time for treatment depends on the underlying etiology, appearance of the scar, location and effect on eyelid closure, and patient preference in some cases. While it may be tempting to intervene early on all scars that appear in the postoperative period, it is important to remember that many may resolve or improve with time and massage. Normal wound healing proceeds through three phases: inflammatory (Days 1-3), proliferative, (Days 4-21), and remodeling (three weeks to two years)^[20]^. Advocates of early laser treatment reason that intervention during the inflammatory or proliferative phases can break this cycle and induce regenerative healing^[21]^. Several studies have demonstrated that PDL when applied as early as immediately following suture removal results in superior scar appearance when compared to no laser treatment^[22–24]^. Because PDL is preferentially absorbed by hemoglobin, its effect is likely greatest when erythema is still present early in the postoperative period. In contrast, scars already undergoing remodeling may respond better to other modalities. A randomized blinded study comparing non-ablative fractional laser (NAFL) to PDL performed at least two months following surgery demonstrated that, in this scenario, NAFL significantly outperformed PDL^[25]^. Interestingly, earlier intervention (within one month) with NAFL appears to offer no significant benefit over observation when re-evaluated beyond one year^3,26^. Moreover, in a prospective randomized control trial evaluating NAFL vs. observation of surgical scars related to direct browplasties performed at our institute, two of eight subjects attributed negative changes in the appearance of their scars to laser treatment, and both of these patients had early intervention (7 and 12 days postoperative vs. 31-767 days)^[27]^. A study comparing ablative laser therapy performed at Postoperative Week 1 to observation similarly found no difference in scar appearance by 12 weeks^[28]^.

## SELECTING THE APPROPRIATE LASER TREATMENT

Provided that the laser light reaches the appropriate depth, how well tissue responds to laser treatment depends on the relative abundance of water, hemoglobin, and melanin (the target chromophores) within the tissue and how selectively each of these molecules absorbs the emitted wavelength. For instance, ecchymoses and erythematous scars may be seen following minor surgery or periorbital trauma. Due to the hemoglobin content within these scars, they respond well to PDL and IPL/BBL™^[29,30]^. Hyperpigmented lesions, especially tattoo related scars and oculodermal melanocytosis (Nevus of Ota), respond well to nanosecond (Q-switched) and picosecond lasers at 694 and 755 nm wavelengths, which are preferentially absorbed by blue/green pigment^[30,31]^.

Scars with greater degrees of hypertrophy (> 3 mm) often require supplemental treatment with more powerful non-ablative lasers^[30]^. For very thick, mature hypertrophic scars and scars under tension, more powerful non-ablative lasers such as Nd:YAG, diode and Er:glass lasers and ablative lasers such as fractional CO_2_ and Er:YAG lasers may be indicated^[32–34]^. Thick scars that cause a cicatricial ectropion may require prompt release of excessive tension due to the risk of ocular damage from prolonged exposure. Both ablative^[2]^ and non-ablative fractional lasers^[19]^ have been used successfully in these cases as an alternative to surgical correction. Although there is a greater risk of complication with ablative lasers, when the energy is fractionated (fractional ablative laser), the risks are lowered. Studies have demonstrated similar effectiveness of fractional CO_2_ and Er:YAG lasers for improving texture, laxity, and dyschromia of the periorbital skin with improvement seen in approximately half of patients by six months^[29]^. One notable instance in which ablative lasers should be avoided, however, is in patients with Fitzpatrick skin types III-VI because of the greater risk of inducing postinflammatory dyspigmentation in this population^[35,36]^. Many providers may opt to avoid laser treatments in these patients altogether; however, certain fractional nonablative lasers, in particular long-pulsed diode, Er:glass, and Nd:YAG lasers, have been demonstrated to be both safe and effective^[37–39]^.

Not all scars contain a predominant chromophore that can be used to achieve sufficient tissue selectivity by changing the laser wavelength. In these instances, tissue selectivity can be achieved by exploiting differences in thermal relaxation times. Ultimately, the goal of treatment is to break down scar tissue, induce neocollagenesis, and stimulate surrounding melanocytes. Both ablative and non-ablative lasers have been used to manage atrophic and hypopigmented scars in periorbital skin that occur in the settings of thermal injury, chemical burns, chronic inflammation, and topical steroid use^[30,40,41]^. Moreover, multiple modalities may be combined in a single session to efficiently address different scar characteristics, e.g., PDL combined with fractional CO_2_ to target erythema and texture, respectively^[42–45]^.

## LASER ASSISTED DRUG DELIVERY

For the most severe scars, lasers may be insufficient to achieve the desired correction. In these cases, laser resurfacing can be combined with intralesional and topical application of antifibrotic agents such as triamcinolone and 5-fluorouracil (5-FU) for enhanced effect^[2,30]^. Laser treatment can be used to create channels within the stratum corneum where topically applied medications can then penetrate deep into the dermis. Fractional photothermolysis further helps to distribute medication across evenly spaced zones. Both erythematous and hypertrophic scars respond well to combination therapy^[2,30]^. For hypertrophic scars, botulinum toxins are increasingly being used in conjunction with steroids and 5-FU to decrease tension and decrease fibroblast activity, although they can only be used in areas where muscle paralysis would not affect eyelid closure (e.g., for medial or lateral canthal scars)^[46]^. In addition to antifibrotic agents, topical application of poly-L-lactic acid and prostaglandin analogs have been used with good effect to treat atrophic scars and improve contour^[47]^ and to enhance re-pigmentation^[48]^, respectively.

## PARAMETERS FOR TREATING PERIOCULAR TISSUE

The key parameters for any laser are wavelength, pulse width, fluence (i.e., energy), spot size, and repetition rate. Wavelength is determined by the lasing medium and filter selection. Pulse width determines the interval of time over which energy is delivered. Fluence is the amount of energy delivered per unit area. In some machines, the total energy is selected. In devices capable of fractional photothermolysis, the energy is further divided into microthermal treatment zones (MTZs). MTZs refer to the number of fractionated spots within a treatment area. Spot size is the diameter of the beam at the surface. Repetition rate refers to the number of pulses per second. Frequently, treatments must be completed over multiple sessions to minimize excess thermal injury and allow for adequate collagen remodeling until the desired result is achieved.

Although periorbital skin is among the thinnest found on the body, higher fluences and higher treatment densities are sometimes used depending on the scar being addressed^[49]^. The rich blood supply to the ocular adnexa facilitates rapid healing from thermal injury^[29]^. Treatment depth can be controlled by adjusting both energy and treatment density. Specific treatment parameters vary by device. For the 2790-nm Er:YSGG laser used in our practice (Pearl Fractional™, Cutera, Brisbane, CA), typical settings may range from 60 to 160 mJ at 4%-12% density^[50]^. For the Ultrapulse Encore fractional CO_2_ laser (Lumenis, Israel), either the Active FX handpiece set at 60-90 mJ and 55%-82% density (Settings 1-3) is used for superficial scars or the Deep FX handpiece set at 8-10 mJ and 5%-15% density is used for deeper scars^[51]^. Higher energy and treatment density with this laser, however, can ablate completely through eyelid skin.

## COMPLICATIONS

Reported complications of laser periocular skin resurfacing include persistent erythema; undesired dyspigmentation; eyelid malposition, viral, bacterial, and fungal infections; burns; corneal injuries; and vision loss. It is imperative that providers adequately inform patients and set realistic expectations. Patients should be advised that they will experience some skin irritation for 24-48 h following treatment. Ablative lasers entail even more downtime. If blistering occurs, generally due to excessive energy or insufficient cooling, the patient should not remove any scabs. Between sessions, energy can be increased by 10%-20% as tolerated or until the desired result is achieved. Patients should be instructed to avoid sunlight between sessions and wear broad-spectrum SPF 30 or higher sunscreen. Sessions should be scheduled approximately four weeks apart to allow adequate recovery. Most laser devices now have built in cooling, but, if this is absent, contact gel should be applied prior to treatment, and the skin should be cooled for 30 min post treatment.

Bulk heating resulting from excessive laser therapy can cause skin damage such as erythema to be as high as 8.8% with CO_2_ laser^[52]^. Scarring and dyspigmentation may also be seen with non-ablative lasers such as Nd:YAG, although less frequently^[29]^. Laser to the periorbital skin presents with the added potential risk of causing harm to the eyes. Ocular structures are highly sensitive to both ablative and non-ablative lasers, but injuries can be entirely prevented with proper eye protection. Although fully occlusive goggles may be sufficient for some cases, more often corneal shields are indicated when eyelid skin is treated. Reported ocular complications include permanent loss of eyelashes and vitreous floaters due to PDL^[29]^; iritis, iris atrophy and posterior synechiae due to IPL; and vision loss in rare cases^[11]^. These complications all occurred when protective goggles were not appropriately placed or were removed to reach periocular skin.

## CASE PRESENTATION

**Figure 2.**
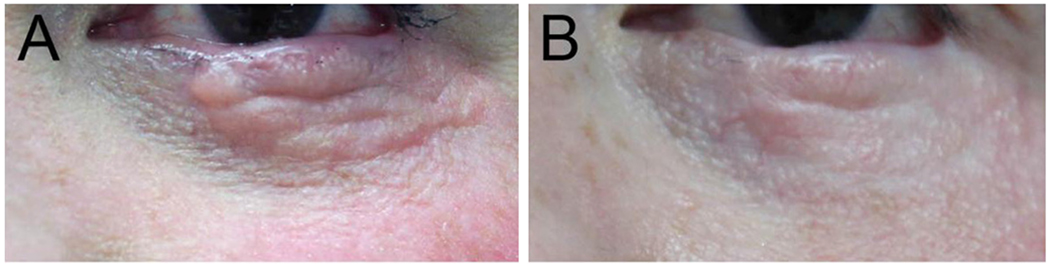
Appearance of a lower eyelid scar from a Hughes reconstruction before (A) and one year after laser resurfacing (B)

A 48-year-old female with a history of left lower lid melanoma underwent Mohs micrographic surgery followed by lower eyelid reconstruction via a Hughes flap and a full thickness skin graft from post-auricular skin. Significant granulation and hypertrophy were noted along the lower lid five months after second stage Hughes without causing significant ectropion. The patient therefore underwent a series of three treatments with 2790-nm Er:YSGG fractional laser resurfacing (Pearl Fractional™, Cutera, Brisbane, CA) spaced 6-8 weeks apart. For the initial treatment, 120 mJ were applied at 12% treatment density followed by a second pass at 80 mJ applied at 8% treatment density. At subsequent sessions, 160 mJ at 12% density were applied followed by a second pass at 120 mJ at 8% density. The patient received prophylactic acyclovir before each session and a corneal shield was placed prior to each laser application. At one year follow up, touch up laser was performed. The patient’s appearance before and one year after laser resurfacing are shown in Figure 2.

## CONCLUSION

Laser skin resurfacing has become an integral tool for the management of periorbital scars. While several studies have demonstrated that early pre-planned treatment with multiple laser modalities can be used to minimize the appearance of postoperative scars, older hypertrophic surgical scars can also respond well to laser treatment, particularly in the periocular region. With the growing number of laser modalities and the capacity to combine laser with topical medications, physicians can tailor treatments to individual skin types and scars. As with all interventions performed around the eyes, a cautious, conservative approach with adequate shielding of ocular structures is recommended to minimize potential complications.

## Figures and Tables

**Table 1.** Properties of lasers used for periorbital skin resurfacing

		Ablative			Non-ablative						
		CO_2_	Er: YAG	Er:YSGG	Er:Glass	Nd:YAG	Alex	Ruby	PDL	KTP	IPL
Wavelength (nm)		10,600	2,940	2,790	1,540	1,064	755	694	585-595	532	500-1200
Fractional		x	x	x	x	x		x	x	x	x
Depth (mm)		2.0	1.0	1.0	1.4	1.0	0.7	0.7	1.2	0.8	
Chromophore	Hemoglobin		x	x	x	x	x		x	x	x
	Melanin						x	x		x	x
	Water	x	x	x	x						
Emission	Continuous	x	x	x	x						
	Long-Pulsed	x	x	x	x	x	x	x	x	x	
	Q-switched					x	x	x			
	Picosecond					x	x				

IPL: intense pulsed light; KTP: potassium titanyl phosphate; PDL: pulsed dye laser

**Table 2.** Depth of periorbital skin

	Epidermis (μm)	Dermis (μm)
Forehead	202	969
Glabella	144	325
Eyelid	130	215
Cheek	145	909
